# The age of intra-African migration: shifting patterns of regional mobility between two global diasporas, 1850–1960

**DOI:** 10.1186/s40878-025-00448-w

**Published:** 2025-05-22

**Authors:** Michiel de Haas, Ewout Frankema

**Affiliations:** Wageningen University, Wageningen, The Netherlands

**Keywords:** Africa, Migration history, Slavery, Forced labor, Refugees, Labor migrants

## Abstract

Rising migration out of Africa is attracting great attention among scholars, policy makers and pundits. In terms of past African mobility, forced emigration through the slave trade, with its nefarious characteristics and long-lasting legacies, has also received much publicity. But what happened to African mobility in the long century between the demise of the trans-oceanic slave trades after 1850 and the gradual resurgence of African extra-continental migration since 1960? This paper adopts the concept of the “Age of Intra-African Migration” to analyze a distinct epoch of widespread and large-scale African mobility, characterized by a succession of overlapping transitions in continent-wide migration patterns. We identify five of these transitions and explain their drivers. Overall, we show that the inward shift of African migration patterns was a consequence of intensified state formation, the demise of the transoceanic slave trades, and export-oriented commercialization. These processes were in turn shaped by trade integration, industrialization and imperialism on a global scale. As such, the Age of Intra-African Migration did not signify a retreat of Africans from global migration altogether, but rather the growing importance of migration destinations across the continent itself. We contend that the ongoing globalization of African diasporas cannot be fully understood without accounting for the dynamics of regional mobility between 1850 and 1960, and that, contrary to popular belief, Africans today are not any more mobile than they were a century ago.

## Introduction

In the four and a half centuries between 1500 and 1950, global long-distance migration was marked by clear geographical divisions. More densely populated regions such as Western and Southern Europe, Northeast Asia (China) and the Indian sub-continent were major migrant sending regions. Lowly populated areas with open land frontiers and exploitable subterranean deposits such as the Americas, Australasia, Southeast Asia and the northern and southern edges of the African continent, were recipients. Under the triple forces of globalization, industrialization and imperialism the land frontiers of these latter areas were extended and their products, mostly minerals and cash-crops, were drawn into global systems of capitalist exchange (Wallerstein, 2004; Barbier, 2010).

Sub-Saharan Africa was an important outlier in this global migratory pattern. Despite comparatively low population densities, vast open land frontiers, and large commodity export potential, it only became a net-immigrant region after 1850. Before 1850, an estimated total of 29 million persons were forcibly taken from Sub-Saharan Africa across the Atlantic (c. 12 million), the Sahara (c. 9 million, a trade that already began in the 7 th century AD), and into the wider Indian ocean basin, mainly from the Swahili Coast and the Red Sea area (c. 8 million) (Austen, 1987, p. 275).^1^ After the ending of the trans-Atlantic slave trades in the 1850s, it would take more than a full century for Africans to reappear in large numbers outside the continent, aside from two major but short-lived periods: the First and Second World War (Killingray, 2022). By this time, global migration patterns had radically reversed however. Long-distance migrations had largely recentered on the industrialized economies in the global North (De Haas et al., 2019). From mid-century onwards, labor migrants from North Africa, displaced during decolonization or invited by European states to accommodate labor market shortages, began to cross the Mediterranean in increasing numbers. North African migrants were gradually and with some lag followed by emigrants from below the Sahara, who followed in the footsteps of much smaller numbers of pioneering students, refugees, soldiers and workers who had already migrated to Europe during the colonial period. Many did not return but settled in Europe, in particular in the former imperial metropoles France, the UK, Belgium, Portugal, Spain, Italy and Germany. North America and, more recently, the Arabian peninsula, also became important destinations (Lucas, 2015). As such, the global diaspora of Africans, which had long consisted predominantly of the descendants of enslaved migrants from West, West Central and Southeastern Africa, has received a major new impetus in the wake of decolonization.

But what defined and determined African migration in the long century between 1850 and 1960? This paper examines the patterns and drivers of migration *within and towards* Africa during what we call the “Age of Intra-African Migration” (De Haas & Frankema, 2022). This distinct epoch in Africa’s migration history, encapsulated between the decline of the external slave trades and the resurgence of extra-continental migration, was a formative period for many contemporary African nations. Migration played a critical role in foundational and spatially uneven processes of abolition of slavery, state formation and export-oriented commercialization. A recent wave of research in economics and political science has shown that such processes have had persistent consequences, but the role of migration has yet to receive focused attention (Müller-Crepon, 2020; Ricart-Huguet, 2022; Roessler et al., 2022; Tadei, 2018).

For our current purpose, we define migration in broad terms, encompassing categories of forced and voluntary migration, circulation and settlement, refugees and labor migrants, both within and between countries, regions and continents. Because migrant categories tend to shift over time, overlap at any point in time, and often reinforce each other, a narrow focus on any one of these migration categories would prevent us from seeing long-run continuities and changes. Moreover, the cross-communal linkages that were created by many different types of migrants–whether they generated anxiety and xenophobia, or creative exchange and solidarity–are key in understanding how migratory changes were linked to abolition, state formation, and export-oriented commercialization *within* Africa.

In defining a distinct Age of Intra-African Migration, we do not want to imply that migration within Africa had been unimportant before 1850. Clearly, the ocean-bound slave trades themselves had caused substantial forced and voluntary migration within Africa, which not only involved soldiers and captives, but also refugee flows and extensive trade diasporas (Curtin, 1984; Lydon, 2009). Other forms of migration linked to trade or religion (pilgrimage) also had deep roots (Usman & Falola, 2009). Neither did intra-African migration become irrelevant after 1960. Even today, the estimated number of Africans that live outside their country of birth but within Africa roughly equals those who live outside the continent.^2^ Below the Sahara, the intra-continental share of migrants is about 70%.^3^ Africans were not entirely absent from the global migration scene between 1850 and 1960, nor were the breaks between these periods sharp and uniform. For example, during the two World Wars several hundred thousand Africans were mobilized as soldiers and porters to support the war effort outside the continent, including the European frontlines, alongside several million of African recruits who were mobilized within Africa (Killingray, 2022, pp. 318−21). We may also refer to people who engaged in long pilgrimage journeys to Mecca (Al-Naqar, 1972), or who, as students, political exiles and in some particularly dehumanizing cases for purposes of entertaining European audiences, found themselves residing or itinerating outside the African continent.^4^ What the concept of the Age of Intra-African Migration instead calls attention to is an unmistaken acceleration and intensification of patterned migration dynamics within and towards Africa, which took place on a continental scale, albeit with numerous regional variations.

## Approach

In this article we construct an narrative on the basis of an in-depth reading of the large but fragmented secondary academic literature produced by African and non-African scholars working in different fields such as history, sociology, economics and political science. We make a conceptual distinction between migration flows, systems and patterns. Building on the pioneering Nigerian geographer Mabogunje (1970), we define a migration system as a web of migratory connections with identifiable nodes that can be demarcated in space and time. We speak of a migration pattern when specific migration systems, or the flows that are part of such systems, emerge simultaneously in different part of the African continent (or any other overarching spatial unit). We make use of data and maps to improve insights into comparative orders of magnitude as well as the timing and direction of specific migration events and flows. These data are originally derived from colonial reports or censuses, as well as (inter)national statistical agencies that collected data on international migration during the post-colonial era. This approach has a number of limitations which can also be considered as challenges for future research. We discuss the three most important challenges.

Firstly, even though we present several estimates of the number of migrants involved to set specific migration events apart from overarching patterns of migration, there is still a lot of research to be conducted to more precisely and extensively measure the size and timing of the many migration flows that have arisen on the African continent in the long century between 1850 and 1960. It is rather ironic that, as a result of the availability of historical sources, the estimates of the number of people forcibly displaced through the Transatlantic slave trade, including their places of origin and destination, tend to be much more accurate than the estimates we have of the migration flows that have occurred *on* the African continent, either at the time of the ocean-bound slave trades or later, when those trades had come to an end. However, there is much more that can be done to quantify intra-African migrations if we were to fully exploit the details contained in (colonial) population censuses, district reports, company records or church registers.

Secondly, as our analytical narrative focuses on defining and understanding the drivers of migration patterns, the lived experiences of persons involved in any of these migrations remain underexposed. Also in this regard, much more can be done. At several key points, we illustrate our narrative through the voices of individual migrants, as documented by contemporaries or scholars conducting oral history interviews. At the same time, we want to stress that a study of continental patterns can only partially be informed by personal testimonies. Migration experiences are deeply influenced by personal conditions and considerations, and while we should be careful not to silence migrants’ voices altogether, we should be equally cautious not to privilege and extrapolate from a selected few.

Finally, a third challenge is that the lion’s share of the sources available to study African migration before 1960 are produced by colonial governments or other agents of imperialism (e.g. European merchants, companies, researchers, missionaries) and not by Africans themselves. This bias in the sources is a given, and the ways to work around these biases are limited. One possible bias that has been investigated by demographers is that colonial censuses systematically tend to underestimate African populations, and such undercounts may, in turn, be more prominent among migrant communities (Manning, 2010; Frankema & Jerven, 2014). This does not mean we should discard these quantitative sources altogether, but implies that we ought to read and interpret them “against the grain” (Stoler 2008).

## The inward turn of forced mobility

The transition that inaugurated the Age of Intra-African Migration involved the decline of the external (ocean-bound) slave trades and the simultaneous intensification of internal slave trading. From the 1750s to 1820s, for every European, four to five Africans disembarked in the Americas (Eltis, 1983, p. 255). In the 1830s the free migration of Europeans numerically overtook the unfree migration of Africans. In the century between 1850 and 1950, an estimated 50 million Europeans settled in the Americas. Of course, the slave trades are a rather distinct form of human mobility. But even though the aspirations and capabilities of the enslaved played hardly any role in their mobility, and even though the trades pre-empted development in the supplying regions, the slave trades are a crucial starting point to understand subsequent migration shifts.

One reason for this is that slavery itself is distinctly migratory, as to enslave a person required the enslaver to take this person out of their home community in order to physically break kinship ties and relocate the enslaved person into an “alien community” with a subordinate social status. This process of alienation is a critical element of what the sociologist Orlando Patterson (1982) has referred to as “social death”. As transporting enslaved people over larger distances reduced chances of flight and facilitated alienation, this raised their value on African slave markets (Lovejoy, 2000, p. 91). Moreover, the internal slave trades required other forms of mobility, which fostered the emergence of spatially extensive trade networks. When enslaved Africans were not sold to European or Arab traders for markets outside Africa, they often became subordinate dependents of an African household which exploited their labor as that of a non-kin outsider. Depending on the context, their children were either born free or inherited slave status. The prevalence of slavery within Africa laid foundations for the first shift we observe, towards a much larger degree of internal deployment of enslaved people.

**Fig. 1:**
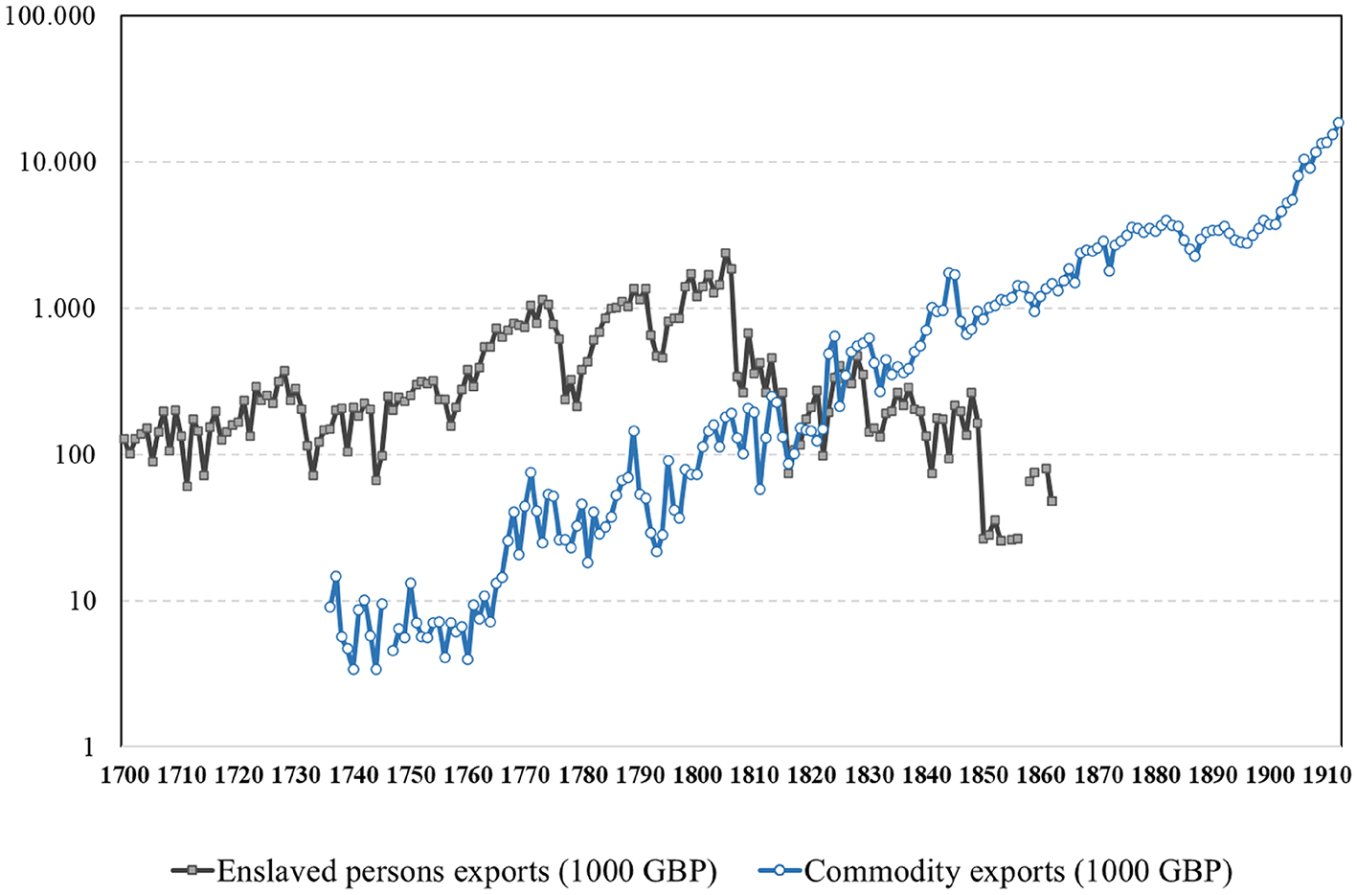
Comparative value of enslaved persons exports and (tropical) commodity exports from West Africa in 1000 GB (log scale), 1700–1913. Source: (Frankema et al., 2018, p. 234)

The inward turn of African migration after 1850 should be understood in a global context of industrialization and trade integration, which greatly expanded demand for a variety of tropical commodities. Rising exports of cash-crops, wood, ivory, livestock products and minerals from labor scarce areas, opened up large spatial opportunity gaps and scope for migration across the Global South. Africa was no exception. Figure 1 shows how the total estimated value of “legitimate” commodity exports from West Africa overtook the value of the “illegitimate” trans-Atlantic slave trades in the 1830s. Historians of Africa refer to this shift from an export trade of enslaved people to agricultural commodities as the “commercial transition” (Law, 1995). This transition depended crucially on local capacities to mobilize voluntary and forced migrant workers to tend the fields, to harvest crops and to transport commodities to the coast or any particular destination inland. Considering Africa’s large ecological diversity, poorly connected interior, scarcity of navigable waterways, and thin populations, the fast growth of output from newly emerging cash-crop zones relied heavily on migrant labor (De Haas & Travieso, 2022). This also applied to the development of gold, diamond and copper mines in Southern, Central and Western Africa (Juif, 2022).

While the export of tropical crops and minerals further grew and diversified under colonial rule after 1880, Fig. 1 shows that exports were already expanding in the second part of the 18th century, involving rising numbers of African farmers, miners, planters and commercial middlemen (Hopkins, 1973; Eltis & Jennings, 1988). Underpinning this African export boom was a major improvement of the terms of trade for African commodities as varied as Egyptian cotton, Nigerian palm oil, Ghanaian gold, East African ivory and cloves, and Senegalese groundnuts. Prices for these goods went up through rapidly growing industrial and consumer demand in the Northern Atlantic and, for most commodities, only hit the ceiling in the closing quarter of the 19th century, when their decline coincided with the European conquest and partition of the African interior (Frankema et al., 2018).^5^

As the trans-oceanic slave trades faded, the raiding of enslaved people to be *retained within* Africa became much more widespread. Slave imports from the Nilotic Sudan into Egypt grew markedly with the expansion of cotton production, especially during the supply disruption caused by the U.S. Civil War, although the share of enslaved people in Egypt remained substantially below some high-density slave societies below the Sahara (Saleh & Wahby, 2022). In East Africa, expanding commodity trades in the late 18th and early 19th century went hand-in-hand with increasing enslavement in the interior to supply the offshore plantations of Eastern Africa: the Mascarenes, Madagascar, and Zanzibar, as well as along the Kenyan coast (Cooper, 1977; Sheriff, 1987; Allen, 2015). Also along the West African coast, where most of the captives were previously embarked for trans-Atlantic shipment, as well as in the interior, the use of slave labor, sourced from vulnerable polities across the region, intensified (Lovejoy, 2000; Austin, 2022). In some places, such as the Sokoto Caliphate, slaves were put to work in large numbers on plantations under conditions reminiscent of chattel slavery in the Americas (Salau, 2018). By the third quarter of the 19th century, one in every five Africans may have been enslaved and in some places this ratio even approached one in two (Coquery-Vidrovitch, 2021; Klein, 1998; Lovejoy, 2000, 2021; Manning, 2021). Although these estimates are rough and tentative, they warrant viewing Africa’s commercial transition as a constituent manifestation of “second slavery”, involving concurrent intensification of slave-based commodity production in Cuba, the US South, Brazil as well as in Sub-Sahara Africa and Egypt (Tomich, 2004; Tomich & Lovejoy, 2021).

The commercial transition also enhanced a variety of forms of non-enslaved migration. Free and dependent workers (e.g. kin, clients, pawns) took part in the cultivation, harvesting, and transportation– e.g. overland head-loading or canoe-transportation– of palm oil, groundnuts, rubber or ivory. Free and enslaved producers often worked side by side, as was the case in the groundnut-producing zones of the Western Sudan and northern Nigeria, and the palm oil producing regions of the Guinea Coast (Manchuelle, 1997; Northrup, 1976), and the lower Volta and Niger basins (Lynn, 1997, p. 51–52). The expansion of commercial agriculture also induced new gender divisions of labor of both free and enslaved men and women, and raised new demand for specialized artisanal skills, military services and traders (Martin, 1988).

Intra-African migrations in this era of commercial transition across Africa were also stimulated by violent processes of state formation, which were partly the result of religious innovation– particularly the invigoration of Islam– and partly due to the emergence of new political constellations and loyalties to stave off European imperial encroachment (Reid, 2012). For example, violent state formation in South Africa, initiated by both Europeans and Africans, resulted in northwards movements by white Boers and militarized Ngoni groups, canonized as respectively the “Great Trek” and the “Mfecane”. These major intra-African migrations also involved enslavement and the gathering of free followers along the way and in places of eventual settlement, and simultaneously resulted in large streams of refugees who feared violence and kidnapping (Wright, 2006). One telling account of the degree of displacement comes from Narwimba, an elderly woman who lived near the Tanzania-Malawi border in the middle of the 19th century and whose story was documented by a German Morovian missionary. While it should be read with care and might be embellished to cater for a sensationalist popular audience in Europe, according to Jacobs (2014, p. 30), her “words resonate as honest and authentic”. Narwimba spent large parts of her life fleeing Ngoni raiders, enslavement and death. At some she was captured, and recalled thinking to herself “now is all over for me. I am far away in a strange land. I shall never see my own country again” (cited in Jacobs, 2014, p. 33). However, she managed to escape, just before being sold to coastal slave traders, and spent her old age in peace, which is one reason why her story survived.

Similar processes of migration, enslavement and displacement took place in the West African savanna, where Islamic empire builders such as Umar Tal and Samori Touré pushed eastwards with their expanding armies (Hanson, 1996; Robinson, 1987). In East Africa, empire building also involved an array of migrations and was tied up with an intensification of the slave and ivory trade. Caravan trades generated a regular supply of enslaved people, often head-loading ivory tusks, from the interior to the planters and merchants at the Swahili coast. The “Mahdi” Muhammad Ahmad, the Nyamwezi king Mirambo and the Afro-Omani Tippu Tip gained control, albeit fleetingly, over large parts of what are today Sudan, Tanzania and Eastern Congo respectively. In the Great Lakes region, the kingdom of Buganda organized raids (Reid, 2012, pp. 117–18; Hakansson, 2022; Pallaver, 2022).

## Migration into Africa

The second transition involved a shift from net-emigration from, to net-immigration to the African continent. Even though this is not “intra-African” migration, the arrival of numerous Europeans and Asians in Africa, was driven by the same processes of abolition, state formation and export-oriented commercialization, and it also further catalyzed these processes and resultant intra-African migration flows. Until the mid-19th century extra-continental settlement in Africa had remained marginal, with the exception of its far northern and southern extremes. However, even in the Cape Colony, the European-descended population had grown to a mere 15 thousand by the late 18th century (Ross, 1975, p. 211). This began to change with the influx of slave descendants who returned to Africa from the 1820s and into the early 20th century, settling (often being settled by their liberators) mainly along the West African coast. Most Afro-American immigrants came from the U.S., Brazil and Cuba in particular and were influential in shaping the urban cultures of cities like Freetown, Monrovia and Lagos (Harris, 1993; Law, 2004; Matory, 1999).^6^ Migration from Europe, the Middle East and South Asia began to rise first towards the more temperate areas, and later into tropical Africa. Asian convict labor and indentured workers settled in considerable numbers in the Mascarenes after the formal abolition of slavery in the 1830s (Allen, 1999, pp. 55–75) to compensate for the loss of slave labor. Attracted by the prospects of cheap land and labor, Europeans settled in the coastal zones of Algeria following the French invasion of Algiers in 1830. The Boers began their treks inland, beyond the borders of the Cape Colony, following the British decision to abolish slave-ownership in all its colonies in 1834.^7^ From the 1850s onwards, the building and operation of the Suez Canal attracted substantial numbers of labor migrants from Europe and the Middle East to Egypt (Carminati 2023).

After 1880, following the partition of Africa, many more newcomers from Europe and Asia settled beyond existing coastal enclaves: free and indentured laborers, traders, farmers, industrial entrepreneurs, mercenaries, missionaries, engineers and colonial officials (Curtin, 1997). South Africa’s white population more than tripled between 1904 and 1970, from 1.1 to 3.8 million, fueled by migrants mostly coming from England (“The Population of British South Africa as shown by the South African Census” 1904; South Africa, 1973). Algeria’s white population increased from an estimated 117 thousand in 1849 (Levasseur, 1856), to 1.1 million at independence in 1954 (Despois, 1956, p. 55). Angola’s white population increased from a mere 10 thousand in 1900 to close to 300 thousand in 1970 (Bender, 1978, p. 20). Large numbers of Indians migrated to East Africa to work on railway construction, and subsequently to settle, mostly as traders. By 1950, close to 300 thousand Asians from today’s India, Pakistan and Bangladesh resided in Kenya, Uganda and Tanzania alone (Trewartha & Zelinsky, 1954, p. 145). Indian and smaller numbers of Chinese indentured workers migrated to South Africa, and free Lebanese to West Africa (Arsan, 2014; Harris, 2010; Dubinsky, 2022). Nonetheless, migrants from outside Africa and their offspring would nowhere make up more than a small minority of the total population of each territory, even in South Africa (less than 20% in 1960, and declining since). Table 1 shows the size and share of non-African settlers in a selection of African countries around 1950. Excluding the largest non-African settler countries such as South Africa, Algeria and the Mascarenes, the combined size of European and Asian communities in tropical Africa remained below 0.5% of the total enumerated population. In comparison, Brazil counted 32 million whites and 14 million people of mixed descent in 1950 (Bender, 1978, p. 21).

Even though the number of immigrants arriving in Africa would remain small compared to New World settler colonies, the continent as a whole shifted from being a net-emigrant to a net-immigrant region. Moreover, the power and influence of non-African settlers reached far beyond their small numbers, as they left deep imprints on the political map of Africa, on state institutions, fiscal systems and transport infrastructures. These immigrants were also important in the spread of Christianity, new forms of Islam, and other elements of cultural exchange (Akyeampong, 2000). European colonization coincided with violent state building practices and a deadly Rinderpest epidemic which caused widespread mobility of people (Sunseri, 2018). Especially during the first decades of colonial rule, European colonizers further catalyzed involuntary mobility, deploying a variety of means to control, or get rid of, unwanted individuals, groups and even entire communities.^8^ Other reasons to displace people in the (early) colonial era included the arrival of white settlers, the creation of game reserves, or campaigns to eradicate diseases such as sleeping sickness (Webel, 2019). Pastoral communities, who used land extensively and generated little revenue for the colonial states, were targeted disproportionally with forced resettlement or restriction of their mobility spaces, amongst others for nature conservation projects (Agrawal & Redford, 2009; Dowie, 2009).

What drove increasing migration into Africa and why did the number of immigrants remain comparatively small? The first question again requires us to consider the abolition of the ocean-bound slave trades and the commercial transition. As Africa’s wealth to the outside world was no longer embodied primarily in exportable human labor, it was to be sought for within the continent itself, through the production of crops and minerals. The latter, in particular, attracted a sizeable numbers of immigrants into Southern Africa. Intensified imperial competition in late 19th century Europe meant that states were willing to provide military, moral and administrative support for such endeavors, culminating in the “Scramble for Africa” during the 1880-1900s (Pakenham, 1992). At the same time, breakthroughs in military technology such as the invention of the machine gun, gave Europeans a definitive military edge over most African societies– Ethiopia defeating Italy in battle in 1896 being the major exception– and made territorial conquest and control of thinly populated lands more viable.

The primary deterrent to large-scale settlement had been the extremely unfavorable disease environment for Europeans in Africa, especially in its tropical and sub-tropical zones. This had made settlement in tropical Africa extremely costly in terms of human life, much more so than in the Asian and American tropics. It was not uncommon for half of all Europeans– missionaries, traders, and soldiers– to die within a year of arrival on African tropical soil. Adult Europeans succumbed to a host of fatal diseases, but especially to the most virulent strain of malaria (*Plasmodium falciparum*) (Curtin, 1989).^9^ Improved medical knowledge in the second quarter of the 19th century, and the wider availability of quinine as an anti-malaria drug, almost literally opened the door to the African interior (Jedwab et al., 2022). By the time that large parts of Africa were brought under colonial control around 1900, the chances of survival of European and Asian immigrants had become much higher.

Still, permanent settlement of European planters and farmers remained largely concentrated in the far northern and southern ends of the continent, which harbored climates that were more familiar to Europeans. These areas were perceived as more agreeable for settlement and allowed for the commercial production of familiar commodities like wheat, wine and sheep’s wool. In tropical Africa, settlement concentrated in the “healthier” highlands, as well as areas with high soil fertility and favorable rainfall patterns, most notably but not exclusively in Southern Rhodesia, Mozambique, Kenya, and parts of the vast Belgian Congo. Contrary to indigenous populations in the Americas and Australasia, African populations (like their South and Southeast Asian counterparts) held up well against European violence and, especially, their pathogens. In fact, the end of the slave trades, the decline of violence, and the gradual spread of new medical infrastructures and technologies enabled Sub-Saharan African populations to stabilize, and eventually to start growing from the 1920s onwards (Manning, 2010; Frankema & Jerven, 2014).

**Table 1:**
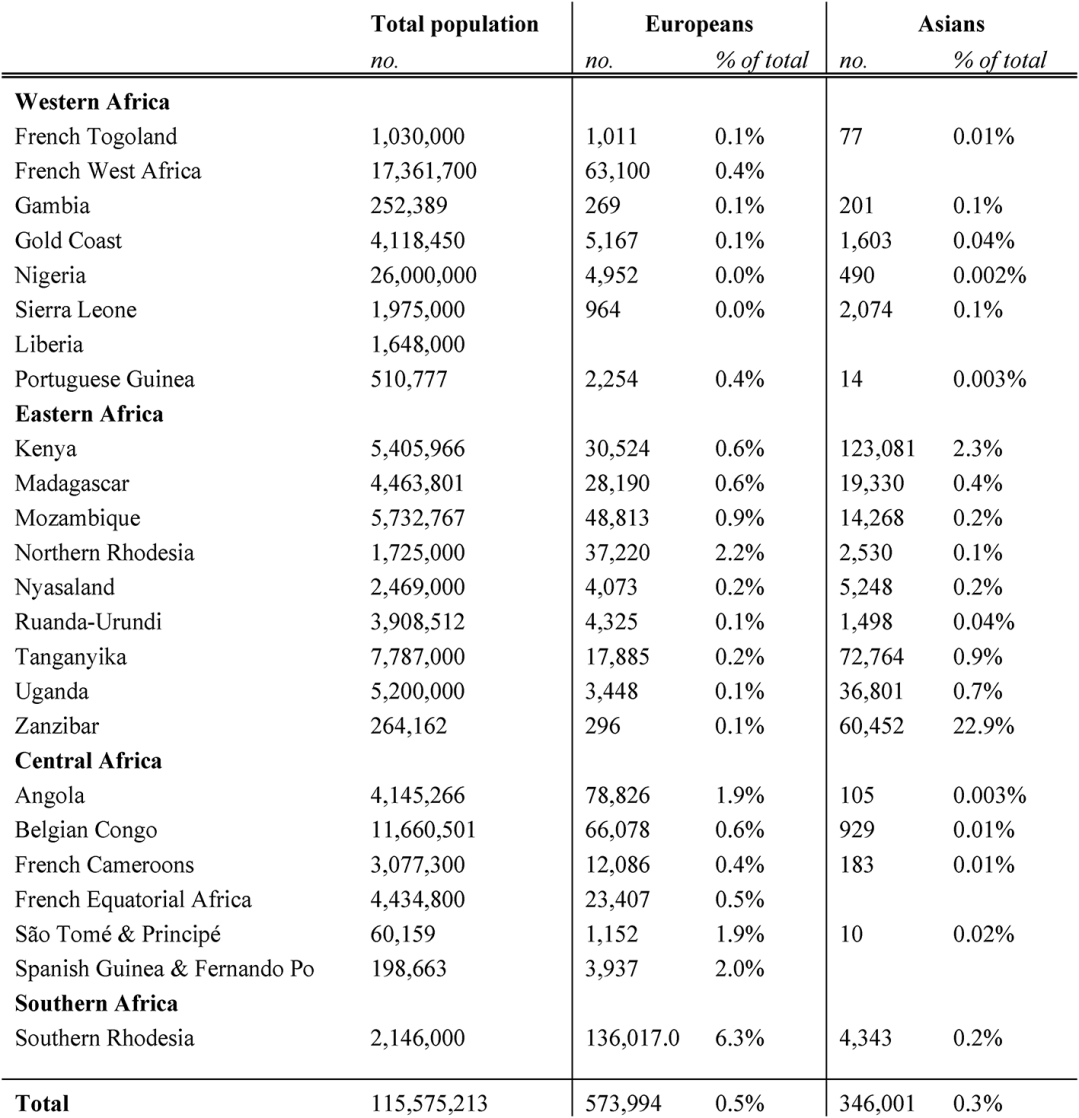
Non-African settlers in selected Sub-Saharan African countries, c. 1950

Source: (Trewartha & Zelinsky, 1954, p. 145)

Note: all observations refer to a year between 1948–1953. Blank cells indicate missing data

Aside from relatively small pockets of European farmers, African farmers kept control over land and their farming activities, and became, in most cases, the backbone of relatively small but fast-growing export-oriented economies were taxed to foot the colonial state formation bill, which involved sizeable European bureaucracies, the proceeds from African cash-crop production were not large enough to warrant the arrival of even larger numbers of European bureaucrats. In fact, colonial states, missionary societies, and businesses increasingly began to rely on– much cheaper– African workers to fill the lower rungs of their organizational ladders. While all these factors thus limited migration into Africa, the transition from a net-emigrant into a net-immigrant region profoundly affected local demographic, socio-economic, religious and political developments, and contributed to the emergence of new forms of intra-African mobility.

## From forced to voluntary labor mobility

A third defining development of the Age of Intra-African Migration was the drawn-out but unmistaken shift from forced to free(er) labor regimes, and concomitant changes in the nature of labor migration. European colonial powers had justified their incursions into the African interior by a mission to “civilize”, which meant the spread of Christianity and putting an end to slave raiding and trading. Stimulating “legitimate commerce” in primary commodities was believed to support that goal (Cooper, 1977; Mann, 2008). Britain and France, the leading colonial powers, had both prohibited the trade and ownership of slaves by 1850. As their empires in Africa expanded, slavery was progressively made illegal. However, rather than being a swift transition, the enforcement of anti-slavery legislation in Africa stretched over a century. Slavery was effectively abolished on the Cape in the 1830s. Elsewhere the practice continued until deep into the 20th century (Miers & Roberts, 1988).

One major reason for the “slow death for slavery”, as Lovejoy and Hogendorn (1993) have put it in reference to the abolition process in Northern Nigeria, was that African elites resisted giving up an institution in which they had large vested interests, and one that had cultural roots in conceptions of reciprocal obligation (labor in exchange for protection and subsistence). Colonial governments, in turn, were wary to provoke unrest, knowing that to rule vast territories on a shoestring, they depended on the cooperation of slave-holding elites. In other cases, territorial control was so tenuous that abolition could simply not be enforced. Economic motives also played a role. In land-rich societies, employers were unable to pay wages high enough to entice peasants away from their farms while also low enough to generate profits, which had been a key factor underpinning the prevalence of slavery in Africa in the first place (Austin, 2009). Colonial states, settlers and concessionary companies often faced similar conditions: low initial profitability of enterprises due to high transportation costs, scattered populations and poor understanding of agricultural conditions. This made their export-oriented ventures, on which colonial revenues heavily depended, unprofitable without tapping into existing slave systems, resorting to forced labor or at least intervening in labor markets to artificially reduce the cost of labor.

When the British conquered the Sokoto Caliphate (Northern Nigeria) around 1900, they encountered an advanced commercial society, which depended on the labor of over 2 million enslaved persons. After an initial wave of mass slave flight into the British controlled Southern Nigerian Protectorate, governor Frederick Lugard adopted a more gradualist approach in the spirit of indirect rule, by collaborating with local elites to retain existing slaves, maintaining Islamic law that permitted the institution of slavery and having slaves pay for their own manumission. Only in the 1930s, and partly as a result of League of Nations scrutiny, did slavery in Northern Nigeria come to a definitive end (Lovejoy & Hogendorn, 1993). In German East Africa, slavery was not abolished at all until Germany’s expulsion from the territory and British take-over during World War I. By this time, however, the prevalence of slavery had already declined substantially, as many enslaved had been able to pursue “emancipation without abolition”, through a range of strategies, often involving migration (Deutsch, 2006).

The French territories were mostly governed under a system of direct rule. As a result, abolition laws had universal and immediate effect in any of the territories under French control. However, *de jure* abolition did not automatically translate into *de facto* slave emancipation. In the 1930s, slavery was still prevalent in less accessible parts of Central Africa that were brought under administrative control after the completion of the Congo-Ocean railway in 1934. In northern Côte d’Ivoire, it took until the late 1940s for many former slaves to finally leave subordinated roles in their masters’ households. Until this time, they had been the ones selected to fulfil colonial forced labor demands, locally or on the plantations in the country’s south (Bassett, 2001, p. 96). In some places of particularly marginal colonial control, such as Mauritania, abolition was never enforced and the practice of slavery persisted into the post-independence era (Ruf, 1999).

Progressive abolition of slavery and emancipation of former-slaves had large and varied repercussions for intra-African migration patterns. Before abolition, most large-scale migration flows consisted of captives who were forcibly taken away from their homelands, or of refugees seeking to avoid capture. At the same time, slavery pre-empted the emergence of large-scale voluntary migration of individuals, as for most ordinary people it was dangerous to move beyond the protection provided by states or local communities (Austin, 2022). With the establishment of colonial control, the fear of capture subsided, although it was, to some extent, replaced by the fear of forced labor recruitment by agents representing, or backed up by, the colonial state (Asiwaju, 1976). Previously captured slaves were able to return home or move on to places where anti-slavery legislation was upheld, economic opportunities existed and stigmas could be shed (Rossi, 2014; Rodet, 2015). Sometimes this led to a mass exodus. Between 1897 and 1907 an estimated 200,000 slaves fled from the Sokoto Caliphate, representing some 10% of the entire slave population (Lovejoy & Hogendorn, 1993, p. 61). In French West Africa, between 1906 and 1911, the estimated number of slaves who departed their former masters upon emancipation exceeded a million (Klein, 1998, pp. 170–4).

The abolition of slavery did not put an end to legalized practices of forced labor. The most extreme and well-known case, also at the time, of labor coercion under the dressing of anti-slavery and civilizing rhetoric was the deadly wild rubber extraction campaign in King Leopold’s Congo Free State (Hochschild, 1998). But there were many other cases. In the Portuguese colonies of Sao Tomé and Principe and Angola, freed slaves were put to work on plantations using recurring five-year labor contracts that were hard to escape (Higgs, 2012). Forced labor schemes were used and justified by colonial governments as an alternative form of taxation in poorly monetized economies (van Waijenburg, 2018). For example, the construction of the Congo-Océan railway in the interwar years involved both voluntary and forced recruitment throughout French West and Equatorial Africa. It cost the lives of an estimated 20 thousand workers as a result of sickness, injury and malnutrition (Daughton, 2021). Forced labor also contributed to forms of migration that were, at least nominally, voluntary, as people sought to find ways to buy themselves out of labor obligations or escape repressive labor schemes, often crossing imperial borders in the process (Allina, 2012; Asiwaju, 1976; De Haas, 2019; Keese, 2016). In some instances forced labor schemes pinned people–and this applied in particular to formerly mobile pastoralist communities–down in specific localities to cultivate local crops or serve white settlers, creating situations of involuntary immobility; in other cases, it involved forced migration to distant places (e.g. to work on railroads or other infrastructure), resulting in involuntary mobility (Okia, 2022).

Large-scale forced labor schemes became increasingly controversial in European metropoles, and especially since extensive media coverage of the atrocities in the Congo Free State. Scandals about the maltreatment of African worker aroused public outcries and the ILO (established in 1921) began to campaign against these schemes. A milestone was the 1930 Forced Labor Convention, which banned signatory nations from using most forms of forced labor. However, ratification was slow in France (1937), Belgium (1944), and Portugal (1956) and many forms of forced labor persisted even after ratification (Keese, 2014; Kunkel, 2018; Okia, 2022). Population growth, which took off during the interwar era, also began to gradually augment the availability of labor, reducing colonial states’ “labor problem”. Mass education, especially provided by Catholic and Protestant missions, and the expansion of apprenticeship systems increased the supply of skilled workers and induced a long-run fall in skill-premiums (Frankema & van Waijenburg, 2023). Moreover, whereas forced labor had been crucial for the establishment of a rudimentary infrastructure of ports, railroads and feeder roads in the early colonial era, the demand for cheap labor attenuated after remote regions had been opened up for export production.

Increasing waves of voluntary labor migrants also resulted in more labor being available at centers of production, thus attenuating spatial disequilibria in labor demand and supply. New transportation infrastructures facilitated their movement, but, in the absence of lorries, trains or ships, many labor migrants travelled distances of up as much as a thousand kilometers on foot. Escaping the threat of forced recruitment at home and acquiring cash to pay taxes played a role in these migration decisions, but they were not the primary motive in most cases (De Haas, 2019; De Haas & Travieso, 2022; Manchuelle, 1997). Instead lucrative export crops made wage labor a viable proposition and migrants often ended up working for other Africans as employees or sharecroppers, or independently on rented land, moving seasonally or for several years (De Haas & Travieso, 2022). Mobility was a response to underemployment and seasonal hunger in the savanna dry season, and served to save up funds to pay for bride-wealth or to complement a meagre family income. Examples of “cash-crop migration” include the large-scale migration of Mossi laborers moving from landlocked, poor and densely populated Upper Volta (Burkina Faso) to the wealthier cocoa producing regions in the Gold Coast (Ghana), and later Côte d’Ivoire (Cordell et al., 1996). One northern Ivorian migrant, who also participated in this migration system and who was interviewed by Thomas Bassett in 1992, recalled about his voluntary migration to the coast that “we would be gone for 6 months, sometimes a year, and come back with a bicycle. Everyone saw that you could earn some money if you left the village, so people left to work for a bike”. He contrasted this clearly to his earlier experiences before the abolition of forced labor: “It wasn’t like that during forced labor; if the workers returned, they were in poor health” (quoted in Bassett, 2001, p. 98). Migrants from Ruanda-Urundi (Rwanda and Burundi), again landlocked, poor and comparatively densely populated, migrated to the cotton- and coffee-producing regions of Uganda and, to a lesser extent, eastern Congo. The story of one returned migrant interviewed by Joseph Gahama in Burundi 1979, illustrates the constraints and aspirations he experienced: “he ‘fled’ to Uganda as soon as he had to buy his first clothes, in order to ‘seek shillings, clothes and bride wealth’. Because he had moved without permission from his chief, his uncle’s cows were confiscated, but he was able to buy new cows and a large piece of land upon return with the clothes he brought back, even though some of them were impounded” (De Haas, 2019, p. 395). Rural-to-rural labor migrants were initially predominantly men who moved on their own initiative. They crossed colonial and imperial borders at will– often moving away from French, Belgian and Portuguese territories, and into British ones. Over time, women also became more migratory, either moving as part of a family or independently. As a result, the gender bias among rural migrants reduced over the course of the colonial era (Cordell, 2013; De Haas, 2019).

Southern, Central and parts of Eastern Africa saw more substantial European settlement and capital investment, and here African migration systems were of a different character. The early cohorts of mine workers in the Congolese Copperbelt were recruited and moved with force, but they were soon replaced by much larger numbers of voluntary and comparatively well-paid migrants who travelled long distances to work in the copper mines, often seeking to avoid harsh and poorly paid labor on colonial plantations (Juif & Frankema, 2018; Juif, 2022). In South Africa and Southern Rhodesia (Zimbabwe) land was alienated on a large scale by European settlers, who needed labor but did not want any, or too many, Black Africans living in their vicinity and on their land. Urban spaces and expanding industrial sectors were also controlled by whites (Frederick & Van Nederveen Meerkerk, 2022; Swanson, 1977). As a result, Black populations were confined to designated rural areas, so-called native reserves, where in situ commercial opportunities were often deliberately curtailed and widespread migration became a near-necessity for survival, let alone obtaining a modicum of prosperity (Arrighi, 1970; Wolpe, 1972). Labor was also recruited on a large scale from neighboring countries, or empires, such as poor, landlocked and densely populated Nyasaland (Malawi) (Groves, 2020), Zambia (Barrett, 2013) and Mozambique (Harries, 1994). Labor laws stipulated that migrant workers should leave their families at home and return after the expiration of their contract (Cordell, 2013; Juif, 2022). Sometimes part of the laborers’ salary was only paid upon return. Returning migrants often took great pride in their experiences. Mr. Maliti, interviewed by Michael Barrett in 2009, repeatedly migrated from Barotseland, Zambia to Bulawayo, Southern Rhodesia, to work as a textile machine operator in the late 1940s. He recalled: “I was buying [things] also for people at home: for my father, my mother, my sisters, nephews, sons and daughters and put them in the suitcase. Even some coats for the parents.– How did you know what to buy?– Mangana! Because I had sense! My wife, I have to dress her! My mother, I have to dress her! My father, I have to dress him! My children, I have to dress them! That’s a personal thing you have to do.” Alongside presents, he brought back “a substantial amount of money”, which he shared among his relatives and also used to buy his first head of cattle (Barrett, 2013, p. 111).

Labor control policies were also significant in reducing settlers’ labor costs in Kenya and Tanzania, but here colonial control over labor and mobility was less comprehensive than in Southern Africa (Fibaek & Green, 2019; Sunseri, 1996). Whereas in most contexts Black migrants’ opportunities for residence in urban spaces increased over time, in South Africa, segregation and the regulation of mobility were tightened further under apartheid (Feinstein, 2005).

In North Africa west of Egypt, labor migration also centered on European settler farms, which had appropriated large stretches of land in the fertile coastal plains, most importantly in Algeria and increasingly in Tunisia as well, to grow grapes and other export crops. European rural settlement displaced local populations who often ended up working as sharecroppers in the expanding mining sector, or in poorly remunerated jobs in fast growing cities. European settler farms also attracted large numbers of Berber labor migrants from densely populated mountainous areas who often migrated seasonally. Migration was most intense towards the Algerian vineyards which attracted 100 thousands of migrants annually by the 1930s. Increasingly, as Algerian Kabyle migrants began to migrate to France from the early 20 century onwards, they were replaced by Moroccan Berbers (MacMaster, 1995; Kateb and Kassar, 2013).

**Fig. 2:**
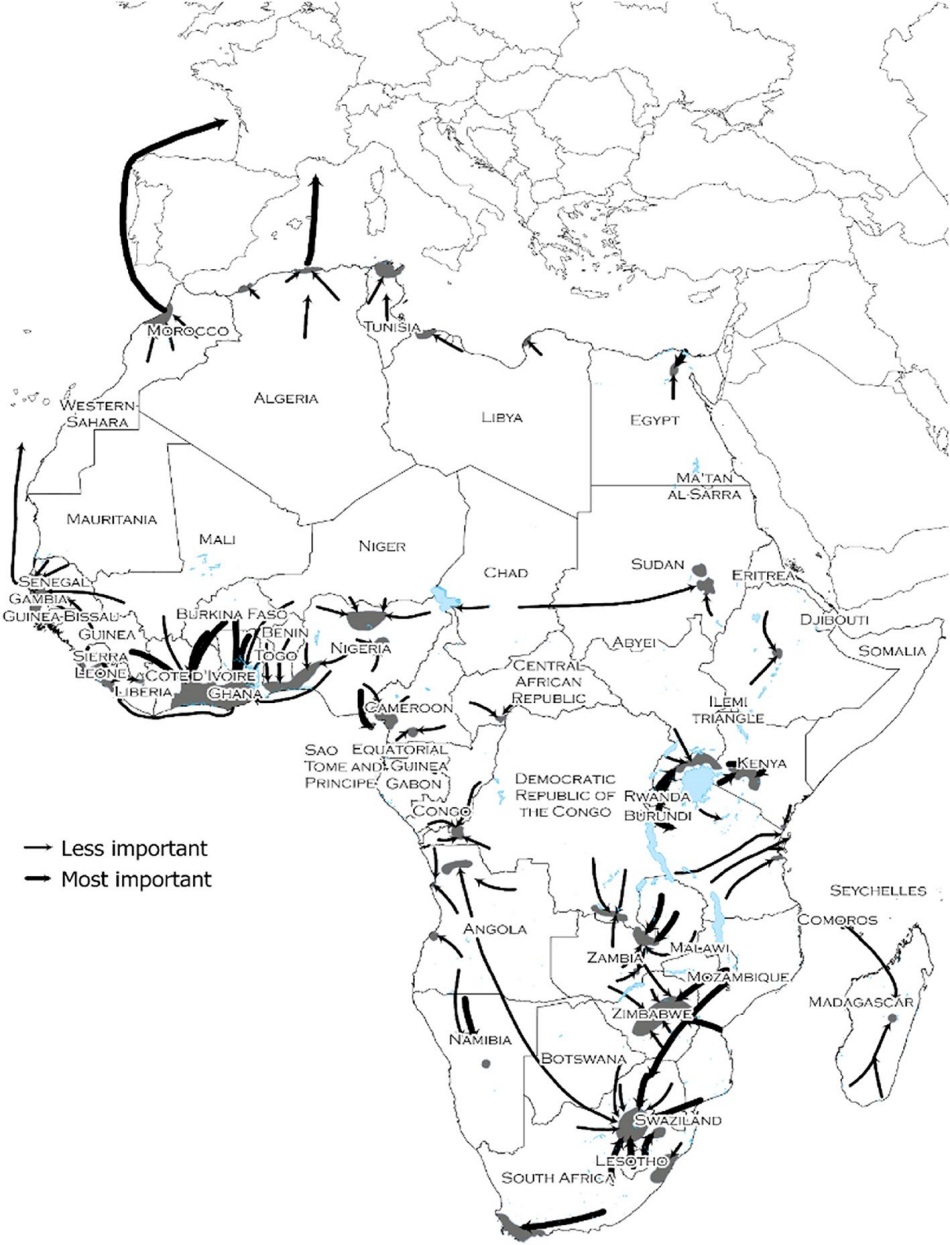
Map of African migration flows and destinations, 1960 s. Source: (Hance, 1970, 147)

By the mid-20th century, across Africa voluntary forms of labor mobility had decisively taken precedence over forced labor regimes. The map in Fig. 2 provides an overview of the most important migrant destination and flows around 1960. Of course, the term “voluntary” is up for scrutiny. As the migrant experienced sampled above illustrate, migratory motives were informed by expectations of better wages, but they could also arise from the sheer necessity to supplement meagre incomes in impoverished sending regions. In any case, it is clear that the size of these labor migration flows was impressive, and most migrants gained substantially from their mobility. In the region west of Nigeria alone the ratio of population in the labor-supplying interior savanna zones versus the coastal cash crop zones shifted from roughly 1:1 to 1:2 between 1920 and 1970 (Amin, 1995, pp. 35–36; Curtin et al., 1995, p. 466). In Buganda, Uganda’s most vibrant cash-crop growing region in the colonial period, over 40% of the total population consisted of immigrants who had arrived in the previous half century, half of whom originated from Belgian African territories (De Haas, 2019). De Haas and Travieso (2022, 238) show that in the 1920s and 1930s many of these cash-crop migrants were able to augment their incomes by a factor of two to four by moving over large distances. For the same decades Frederick and Van Nederveen Meerkerk (2022) find that in the major cities of the Belgian Congo unskilled day laborers could earn three times as much as in the countryside. Ribeiro da Silva and Alexopoulou (2022) record income gaps of a factor four to six between Mozambique and the South African Rand areas. African mine workers’ real wages on the Central African Copperbelt underwent a striking sixfold increase over the colonial period (Juif & Frankema, 2018). Such gains were comparable, sometimes even higher, than those of Europeans migrating to the Americas, or Indians and Chinese moving to Southeast Asia during the Age of Mass Migration (Hatton & Williamson, 1998, 2005, pp. 136–37).

## From rural to urban destinations

A fourth major transition in African mobility patterns that emerged during the Age of Intra-African Migration was the shift from predominantly rural to urban destinations. While cities have long existed and played important roles across African societies, until the mid-20th century, the majority of intra-African migrants, especially outside of South and North Africa moved between rural areas. In Eastern Africa, the urban share of the population stood at a mere 5.7% in 1950, compared to 9.3% in West Africa and 13.9% in Central Africa (United Nations, 2018). After 1950 urban destinations quickly gained in relative and absolute importance (Frederick & Van Nederveen Meerkerk, 2022; Meier zu Selhausen, 2022). In most of Northern and Southern Africa, migration to urban areas was already much more advanced by this time. In Northern Africa, 25.9% lived in cities in 1950, and in Southern Africa the share was as high as 37.7%. South Africa’s high urban share (42.2%) had its roots in the late 19th century diamond and gold booms. Continent-wide urbanization rates rose from c. 11% in 1950 to c. 23% in 1980 and surpassed 40% by 2020 (United Nations, 2018).

In many cases, the colonial era was foundational to African urban growth. Cities such as Ndola and Lubumbashi started out as mining towns. Abidjan, Accra, Dakar, Kampala and Lagos grew as trade hubs for cash-crop exports or as colonial capitals. Some of the largest cities today, such as Nairobi, sprang up along newly built railroads in areas that had been thinly populated (Jedwab & Moradi, 2016). Urbanization rates remained low for long, not only because rural areas were vibrant and urban employment limited, but also because colonial authorities restricted permanent urban settlement and promoted circular migration. This control of urban spaces, often through racial segregation, was more common in Central, Southern and Eastern Africa than in Western and Northern Africa, where large cities had been more firmly established already in the pre-colonial era. In most cases (apartheid South Africa being a major exception), restrictions on Black urban settlement were loosened over time, especially from the 1940s onwards (Coquery-Vidrovitch, 2005). Expanding commerce, educational opportunities and new consumer products and technologies enhanced the demand for diverse unskilled and skilled jobs, which in turn attracted a growing number of rural migrants to the cities (Meier zu Selhausen, 2022). The shift from rural to urban destinations also resulted from the foreclosure of opportunities in rural areas, as land frontiers began to close, late colonial and early post-colonial states redistributed resources from agriculture to urban infrastructures and industries, and cash-crop booms eventually turned to bust (De Haas & Travieso, 2022).

As had been the case with rural-rural migration, the gender composition of rural-urban migration also underwent major changes. Initially, urban migration was heavily male-dominated. In later phases, women increasingly joined their husbands in cities, or migrated to cities independently, stimulated by expanding opportunities of both formal and informal service sector employment, urban health care and schooling (Juif & Frankema, 2018; Obbo, 1980). This transition was most pronounced in Eastern and Southern Africa, where male and female jobs tended to be more strictly separated, and work outside the home remained a male prerogative for a much longer time. The story of Filo provides one particular window into how this transition was experienced:

“My husband and I decided to sell the land and buy a small plot near Kampala. In 1964 we bought an acre, had a small garden and built houses on the rest. He is now a car mechanic and he also repairs bicycles and motorcycles. I am still a hairdresser and a bride-dresser and a dressmaker. I like it here very much. We had to migrate from the village because the women were so jealous of my prosperity that some accused me of having bought witchcraft that enabled me to prosper at their expense. […] I wanted much more out of life than digging. I wanted to learn how to read and write. The world was changing and leaving the women behind” (Obbo, 1980, pp. 73−4).

**Fig. 3:**
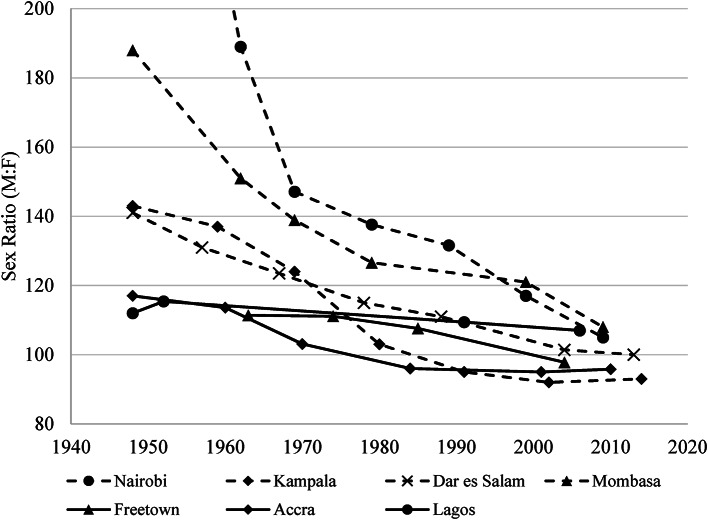
Sex ratios in East and West Africa’s major cities, 1948–2015. Notes: A sex ratio of 100 means an equal number of men and women. West African cities are indicated with solid lines, East African cities with dotted lines. Source: (Meier zu Selhausen, 2022, 291)

In the older West African cities, women had long been involved in retail trading, and had a greater presence in cities earlier on (Meier zu Selhausen, 2022). As Fig. 3 shows, in major cities across West and East Africa, the sex ratios, which are defined as the number of men per 100 women, have all converged to parity.

African cities provided real opportunities for migrants to fulfil a wide array of aspirations, economic and otherwise, and they continue to do so today. However, they did not turn out to be hubs of salaried employment in an expanding formal, industrial sector. Rather, migration to cities involved the growth of large informal sectors in which underemployment is rife and competition for jobs cut-throat. At least partially as a result of this, the relative importance of migration as a driver of urban growth has declined. Up to the end of the 20th century most African cities grew primarily as a result of rural in-migration. However, by the 2010s, natural increase had become the dominant factor in urban growth (Meier zu Selhausen, 2022). Continued urbanization today is thus driven primarily by the endogenous growth of a relatively young population, although rural-urban and, increasingly, urban-urban migration still plays a substantial role as well.

## From intra-African to global migration

**Fig. 4:**
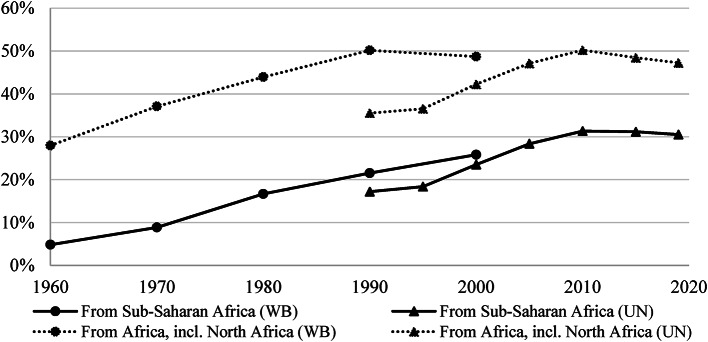
Share of international migrants from Sub-Saharan Africa and Africa (incl. North Africa) that have moved outside the continent, 1960–2020. Notes: The fact that the extra-continental shares are lower according to the UN estimates is partially due to the fact that UN data fully accounts for refugees, a larger proportion of which remained within the continent.Sources: Population data from the World Bank (https://databank.worldbank.org/source/world-development-indicators). Emigrant stock from the World Bank (https://databank.worldbank.org/source/global-bilateral-migration) and the United Nations (https://www.un.org/en/development/desa/population/migration/data/estimates2/estimates19.asp). Refugee stock from UNHCR (https://www.unhcr.org/data.html).

The final, fifth, transition in migration pertains to the end of the Age of Intra-African Migration: a gradual and partial shift from intra-regional to extra-continental destinations. Although this shift cannot be pinned precisely on one historical moment, and remains far from complete today, the fact that African migrants–in their aspirations and actual migration decisions– increasingly aim for extra-continental destinations marks the end of the Age of Intra-African Migration. As Fig. 4 shows, in 1960 just over 1 in 4 Africans and only 1 out of every 20 Sub-Saharan Africans who lived abroad did so outside of the continent. By 1990, 1 out of 2 international migrants from Africa as a whole, and 1 out of 5 from Sub-Sahara Africa, resided outside the continent. In 2020, 1 out of 3 Sub-Saharan migrants resided outside the continent. Not only a much larger proportion, but also a larger number of Africans ended up living outside the continent. Whereas in 1960 only 13 out of every 10,000 people born in Africa were based outside the continent, this figure had increased to 81 per 10,000 by 2019, implying a six-fold relative (per capita) increase and, when we factor in fast overall population growth, a 30-fold absolute increase. From North Africa, nearly all international migration has been extra-continental, with Europe and the Arabian Peninsula as prime destinations. Below the Sahara, most inter-continental migration arose from coastal nations including Ghana, Kenya, Nigeria, Senegal, and South Africa, as well as Ethiopia, each of which has the majority of its emigrants living outside Africa (Natale et al., 2018, p. 11). Migrants from other African nations remain mainly focused on intra-continental destinations.

What drove this partial shift in migratory destinations from within to outside the continent? We argue that shifting opportunities in different potential receiving regions were decisive. Many of the spatial opportunity gaps that had emerged in Africa since the 19th century began to narrow in the second half of the 20th century: labor markets had become saturated because of an expanding supply of skilled and unskilled workers, rising education levels and rapid population growth (Hatton & Williamson, 2003). Moreover, with some exceptional destinations, such as Côte d’Ivoire (during the heydays of its postcolonial cocoa boom) and South Africa, spatial opportunity gaps of the order seen in the colonial era had largely disappeared by the 1970s, and have not reappeared since. Opportunities for the growing numbers of educated Africans were declining, as evidenced by rapidly falling skill premiums (Frankema & Van Waijenburg, 2023). In the 1970s and 1980s, worsening terms of trade for export commodities and mounting government debt positions led to widespread economic crises (Bates, 2008). Many colonial export-producing enclaves collapsed altogether. In cases where spatial opportunity gaps continued to exist, receiving communities increasingly saw immigrants as competitors for land and jobs. In South Africa, where income levels far exceed those of neighbouring countries such as Lesotho, Malawi, Mozambique, and Zimbabwe, labor immigrants have met with rising hostility, especially after 1994 (Crush, 2000; Lucassen, 2022).

These signs of changing attitudes towards immigrants entailed a shift in public perceptions of the value of migrant labor and the presence of “strangers” in the context of anti-colonial nationalism, and a growing political emphasis on formal citizenship and territorial borders, even though the latter typically have remained porous (Fernandez, 2013). Many of the newly sovereign states of Africa thus adopted (much) stricter immigration acts than their colonial antecedents. In the wake of economic crises, African governments also began to expel undocumented labor migrants. These migrants had sometimes lived for decades across the border, never having been required to have a residence or work permit before (Frankema, 2022). In some cases mass expulsions involved hundreds of thousands of people at once, the expulsion of between half and one million West African migrants from Ghana in 1969, and even larger numbers from Nigeria in 1983 and 1985 being the most extreme cases (Adepoju, 1984; Bredeloup, 1995).

To interpret this shift, Robin Cohen has made a distinction between absorption and expulsion as “two forms of engagement with strangers—the *anthropophagic*, where outsiders are swallowed and digested, and the *anthropoemic*, where aliens are discarded, institutionalized, incarcerated or expelled” (Cohen, 2019, p. 45). In the long course of history, slow but definitive changes in population densities underpinned this momentous shift, which is closely tied to the relative value of human labor, and especially low-skilled labor. Whereas enslaved migrants in pre-colonial times, and forced labor migrants in the colonial era, were forcibly taken towards the main centers of economic, social, military and cultural activity, in the post-colonial era people were increasingly sent away and repatriated– or isolated in the case of refugees who could not be denied access under international law (Frankema, 2022).

As opportunity gaps were closing within Africa, through economic, environmental, and social changes or political regulations, Africans increasingly chose destinations outside the continent. At the same time, migration towards Africa also declined, and even reversed, in the wake of decolonization. Many settlers and their descendants were forced to emigrate or remigrate to Europe or Asia. The conclusion of the Algerian war of independence (1954–1962) led to a mass exodus of *Pieds-Noirs*. Indian settler communities in Uganda, and to a lesser extent other East African nations, dwindled as a result of mass emigration under threat of growing hostilities, expropriations or even an outright ultimatum instigated by the Amin regime. The lion’s share of the circa 90,000 Belgians left the Congo in the early 1960s. More recently, white settler farmers in Zimbabwe who gave up under threat of land reforms either moved to South Africa or chose an extra-continental destination.

But the growing importance of extra-continental destinations was not just driven by changes on the continent. Post-Second World War Western Europe had embarked upon a “golden age” of industrial catch-up growth (Eichengreen, 2007), resulting in increasing shortages of low-skilled labor during the 1960s and 1970s, especially in the northwestern parts of the continent. Tight labor markets were relieved by migrants, initially from Southern Europe, but increasingly from North Africa as well. As the numbers cited above already illustrate, the number of Africans from below the Sahara migrating out of the continent was much smaller at this time, but not negligible. The late colonial period had already seen a rise in the number of Africans studying and working in Europe, and this number grew further in the 1960s. When European migration policies towards formerly colonized people became more restrictive and African countries began to experience rising instability and economic collapse during the 1970s and 1980s, many of these earlier migrants decided not to return to Africa and began to form substantial diaspora communities in Europe and, increasingly, the United States (Lucas, 2015). Refugees from conflict areas in the Horn of Africa, Central Africa, Nigeria and other places joined their ranks, although it is important to note that, just as today, the overwhelming majority of refugees remained within their home region (Frankema, 2022). The economic depression of the 1980s in Europe and the United States smothered a period of moderate openness towards African migrants, and migration policies have tightened significantly since. However, continued chain migration and family reunion has sustained and expanded existing diasporas. Moreover, demographic decline, ageing, and a widespread reluctance to engage in certain type of occupations by wealthier Europeans gave the demand for cheap immigrant labor a more structural character (De Haas, 2008).

Initially, the linguistic, political, and economic ties of African countries with former colonizers played an important role in the choice of destination. Specific regions and ethnic groups were notably overrepresented among these early migrant workers in Europe, such as the Kru and Yoruba in Britain and the Soninke in France (Frost, 2002; Harris, 2006; Manchuelle, 1997). Especially migrants from former French and Portuguese colonies benefitted from “bridgehead communities” in their former metropoles (Lucas, 2015). In the 1950s there were only 2 thousand registered Black African workers in France, mostly from Senegal and Mali. By the mid-1990s this number had risen to 60 thousand, still predominantly from the same regions (Manchuelle, 1997, p. 2). Even in 2019, over half of all recorded African extra-continental emigrants from former French colonies were living in France.^10^ In recent decades, however, increasing shares of African migrants have moved to other destinations. In the oil-rich and wealthy, but sparsely populated Gulf economies, demand for Asian and African migrant workers also surged. In this case Africans migrate and work under strictly temporary and severely controlled conditions. For example, 480 thousand Ethiopians were recorded to have migrated to the Middle East through legal channels between 2008 and 2014, of which about 4 in 5 to Saudi Arabia. Considering that for every Ethiopian who migrates to the Gulf countries regularly, there are two irregular migrants, the total number of Ethiopian migrants is likely to be much greater (IOM, 2020b, p. 7). Largely as a result of tightening restrictions along Europe’s Mediterranean borders, North African countries, and Libya in particular, have hosted large and more permanent Sub-Saharan African communities (Di Maio et al., 2023).

The diversification in non-African destinations has been facilitated by further declines in transportation and transaction costs. Access to mobile and digital information and communication technology, has made it much easier to gather information, to remain in touch with “home”, and to organize multiple attempts to cross fortified borders, through regular and irregular means, in the hope that one time will be successful. For those using regular (i.e. legally recognized) migration routes, the journey itself became shorter and easier to plan with airlines maintaining regular connections. International cash transfers to support migrants’ families back home with remittances became very easy. With the continuing rise of education levels after independence, an increasing share of Africa’s young generations is qualified to migrate to rich countries to study or take up jobs. Indeed, migrants who depart the continent tend to be more educated and less likely to be male compared to intra-African migrants (Allie et al., 2021). More advanced education at the country level has also been found to correlate with “intercontinental migration [of Africans] but not [with] shorter movements within Africa” (Lucas, 2015, 1496).

Irregular attempts to cross the Sahara, Mediterranean and Red Sea often happen under appalling conditions, involving abuse and extortion by human smugglers, violent responses of border police and other enforcers, who face little accountability for their treatment of migrants, and exposure to the vagaries of weather and the sea. Despite these hardships, however, many continue to “scale the fences” (UNDP, 2019). While the rise of this type of African immigration into Europe and the Arabian Peninsula may appear to be a new phenomenon, parts of it closely mirror intra-African migrations in earlier times. For example, an increasing number of labor migrants from rural areas in West Africa (e.g., Senegal, Mali, Niger) find work as seasonal workers in Southern European agriculture, with or without legal work permits (Hoggart & Mendoza, 1999), or in poorly paid jobs in catering and cleaning services in European cities.

How should we then view the post-1960 rise of African extra-continental migration in a long-term historical perspective? In most respects, extra-continental migration from Africa after 1960 has little in common with earlier trans-oceanic enslaved migrations before 1850. The renewed presence of Africans on the global migration scene is now largely the result of decisions made by migrants themselves, seeking opportunity, fleeing from conflict and even expulsion, or both. Enslaved migrants were typically cut off permanently from their ancestral homelands, even though some managed to return to Africa, and contribute to vibrant immigrant communities across the continent. Post-colonial migrants, instead, have kept close ties with sending communities, building international diasporic networks, sending hard-earned remittances to families and communities in sending regions, and sometimes returning home after the expiration of labor contracts or failed asylum attempts. Meanwhile, migration out of Africa today has much more in common with voluntary migrations within Africa a century ago than is often acknowledged. In the early 20th century migrants would walk for weeks to reach their destination, often under pitiful and hazardous circumstances. The major difference today is that migrants are able to bridge larger barriers and distances with a comparable level of risk and distress. Borders and passports have become more important, but the journeys have remained equally hazardous, and work conditions equally harsh. Destinations have changed, and new information and communication technologies have compressed physical distances. However, migration decisions today have as much to do with migrants’ aspirations and their awareness of spatial opportunity gaps as they had a century ago, when intra-regional mobility prevailed over extra-continental moves.

## Conclusion

We have sought to define the Age of Intra-African Migration which unfolded during the long century between circa 1850 and 1960, and understand the drivers of shifting migration patterns that occurred during this period. We have identified five overlapping shifts which unfolded on a continental scale, albeit with notable regional variations and differences in timing. The first shift, involving an inward turn of forced mobility directly connects to the demise of the transoceanic slave trades. The final shift links the Age of Intra-African Migration to Africans’ increasingly global migration in recent decades. Two of the in-between shifts from forced to voluntary labor mobility and from rural to urban destinations form crucial links in this long-term migration trajectory. Even though most migration unfolded within the continent, the Age of Intra-African Migration should be understood in a global context, and was driven by global processes of trade integration, industrialization and imperialism. Whereas Africa’s position in such global processes had for centuries revolved around the export of enslaved persons, the continent now became an important frontier of colonial state formation and export-oriented commercialization. As a result, numerous attractive migration destinations emerged, to which not only Africans but also settlers from Europe and Asia responded. Receiving regions and societies across the continent, moreover, proved to have a large absorptive capacity, and while this, in many cases, was a transitory phase, the large and mostly voluntary migration flows this engendered laid crucial foundations for the new African diaspora’s that have emerged since the 1960s. Considerable scope for further research on the Age of Intra-African Migration remains, both in deepening our understanding of patterns in migrants’ motivations and experiences, and in further quantifying migrant flows, systems and patterns across the continent.

## Data Availability

The datasets used and/or analysed during the current study are available from the corresponding author on reasonable request.

## Footnotes

1 The trans-Atlantic trades came to a definitive end in 1866. Nine out of ten persons who were taken via the Atlantic corridor did so in the two centuries between 1650 and 1850 (Slave Voyages v2.2.13). Slave exports in the Northern part of the Indian Ocean continued until the early 1870 s, and illicit trading only ended in the 1890 s, but in much smaller numbers. Moreover, an estimated 50% of these slaves were put to work on clove plantations along the Swahili Coast (incl. Zanzibar and Pemba) and were thus retained within Africa. The same applies to the East African slave exports to the Mascarenes, which ended in the late 1820 s (Cooper, 1977; Allen, 2015).

2 For comparison, only 15% of Latin American and Caribbean international migrants stay within their respective regions (Lucas, 2015, 1448).

3 Statistics on shorter-term circulation and intra-African flows are too poor to generate any precise estimates (IOM, 2020a), but if we were to consider circulatory flows, the proportion of intra-African movements in total migration will likely be even larger.

4 There is a growing literature documenting Africans’ global migration beyond the slave trades. On the longue durée history of African presence in Europe see Otele (2020).

5 The terms of trade for African commodities then underwent a structural decline up to the late 1930 s, but after 1900, the losses in relative value were more than compensated for by rapidly expanding output volumes. The range of export crops widened and new mining zones opened up, first in South Africa following discoveries of diamonds (1866) and gold (1886), later in many other places of Southern, Central and Western Africa.

6 Freed slaves from ships intercepted by British squadrons also disembarked in both countries, but these may be better referred to as intra-African migrants, albeit of a rather peculiar category.

7 Trade in slaves had been prohibited earlier, in 1807/8, but this had left slave-ownership untouched.

8 The genocide of the Herero and Namaqua in German South West Africa (Namibia) in 1904-8 provides a flagrant example of the latter. Note that 19 th century African state-building processes typically involved attempts to acquire new followers, either free or enslaved, while in Namibia indigenous people were expelled or exterminated.

9 Other major killers were yellow fever, typhoid fever, and gastrointestinal infections.

10 3.4 out of 6.8 million according to UN data (see Fig. 4 for the source).
